# Rapid antiretroviral therapy initiation in patients with advanced HIV disease: 6-month outcomes of an observational cohort evaluation in Lesotho

**DOI:** 10.1371/journal.pone.0292660

**Published:** 2023-10-11

**Authors:** Appolinaire Tiam, Heather Paulin, Rhoderick Machekano, Ikwo Oboho, Elfriede Agyemang, Fred Asiimwe Mugyenyi, Llang Maama-Maime, Yohannes Mengistu, Tsitsi Chatora, More Mungati, Majoalane Mokone, Tsietso Mots’oane, Annah Masheane, Vincent Tukei

**Affiliations:** 1 Elizabeth Glaser Pediatric AIDS Foundation, Washington, District of Columbia, United States of America; 2 Centers for Disease Control and Prevention, Atlanta, Georgia, United States of America; 3 Centers for Disease Control and Prevention, Maseru, Lesotho; 4 Ministry of Health Lesotho, Maseru, Lesotho; 5 Elizabeth Glaser Pediatric AIDS Foundation, Maseru, Lesotho; Management Sciences for Health (MSH), ETHIOPIA

## Abstract

For adults and adolescents, the World Health Organization defines advanced HIV disease (AHD) as a CD4 (cluster of differentiation 4) count of <200 cells/mm^3^ or a clinical stage 3 or 4 event. We describe clinical outcomes in a cohort of AHD patients at two regional hospitals in Lesotho. From November 2018–June 2019, we prospectively enrolled eligible patients (≥15 years) not on antiretroviral therapy (ART) presenting with WHO-defined AHD into a differentiated model of care for AHD (including rapid ART initiation) and followed them for six months. All patients received Tuberculosis (TB) symptom screening with further diagnostic testing; serum cryptococcal antigen (CrAg) screening was done for CD4 <100 cells/mm^3^ or WHO clinical stage 3 or 4. Medical record data were abstracted using visit checklist forms. Categorical and continuous variables were summarized using frequencies, percentages, and means, respectively. Kaplan-Meier was used to estimate survival. Of 537 HIV-positive patients screened, 150 (27.9%) had AHD of which 109 were enrolled. Mean age was 38 years and 62 (56.9%) were men. At initial clinic visit, 8 (7.3%) were already on treatment and 33% (36/109) had presumptive TB per symptom screening. Among 39/109 (40.2%) patients screened for CrAg at initial visit, five (12.8%) were CrAg-positive. Among 109 enrolled, 77 (70.6%) initiated ART at their initial clinic visit, while 32 delayed ART initiation (median delay: 14 days). Of the 109 participants enrolled, 76 (69.7%) completed the 6-month follow-up, 17 (15.6%) were lost to follow-up, 5 (4.6%) transferred to other health facilities and 11 (10.1%) died. The 6-month survival was 87.4%; among 74 patients with a viral load result, 6-month viral suppression (<1,000 copies/ml) was 85.1%. Our study found that even after the implementation of Test and Treat of ART in 2016 in Lesotho, over 25% of patients screened had AHD. Patients with AHD had a high prevalence of TB and CrAg positivity, underscoring the need to assess for AHD and rapidly initiate ART within a package of AHD care for optimal patient outcomes.

## Introduction

The World Health Organization (WHO) defines patients presenting with advanced HIV disease (AHD) as having a Cluster of Differentiation 4 (CD4) cell count of <200 cells/mm^3^ or WHO clinical stage 3 or 4 disease [1]. Achieving immune system recovery with antiretroviral therapy (ART) is the primary means to reduce morbidity and mortality related to HIV-associated opportunistic infections (OIs) and delays in ART initiation result in avoidable deaths [2,3]. Early mortality among persons with advanced immunosuppression after starting ART not only reflects the spectrum of causes of death prior to ART initiation but in some cases, is also the result of immune reconstitution inflammatory syndrome (IRIS) [4]. Strategies to reduce early mortality and morbidity rates among people living with HIV (PLHIV) presenting to care with AHD include earlier OI diagnosis, prophylaxis and treatment, timely ART initiation, and close follow-up and monitoring [5–9].

The WHO recommends differentiated HIV care, tailored to the unique needs of different patient populations, that includes a package of care for patients presenting with AHD focused on preventing death and reducing morbidity detailed below [1,5–7]. The AHD package of care includes: rapid initiation of ART (within 7 days) in the absence of contraindications, screening for active TB disease with TB treatment or TB Preventive Treatment (TPT) as indicated, cotrimoxazole prophylaxis, cryptococcal antigen screening (CrAg) for those with CD4 cell count <100 cells/mm^3^ (with additional consideration for CD4 <200 cells/mm^3^), treatment for cryptococcal meningitis (CM) or preemptive fluconazole therapy for those with cryptococcal infection without CM, and intensive follow-up. Despite the strong evidence base for the key interventions included in the WHO recommended package of care for PLHIV with AHD, implementation and coverage of these interventions in many countries remains suboptimal to date [1,10–14].

With an estimated HIV prevalence of 21.1% among adults aged 15–49 years, Lesotho has had the second-highest HIV prevalence in the world for many years [5,15]. In 2015, it was reported that there had been minimal change in the median CD4 cell count of approximately 200 cells/mm^3^ at enrollment in care over the years in Lesotho [16]. In alignment with the WHO recommendations, the country adopted the Test and Treat policy in 2016. All PLHIV are eligible for ART regardless of their CD4 cell count or HIV clinical stage to improve ART coverage and reduce morbidity and mortality among PLHIV [5]. A substantial proportion of patients may continue to present or re-present for HIV treatment with AHD, until other complex barriers to earlier HIV diagnosis and prompt linkage to treatment are overcome [17]. While the Government of Lesotho had endorsed the WHO AHD package as part of differentiated AHD care services and the comprehensive test and treat guidelines, in practice, the AHD interventions were not systematically implemented nor adequately scaled up [5]. Elizabeth Glaser Pediatric AIDS Foundation (EGPAF), with Centers for Disease Control and Prevention (CDC) support, provided technical assistance to the ART program in selected health facilities to facilitate the implementation and integration of the AHD package of care.

This study evaluated the clinical outcomes (retention and viral load suppressions) in a cohort of patients with AHD accessing care in these programs after the above AHD package of care was implemented within routine differentiated care services.

## Materials and methods

### Study design

We prospectively enrolled a cohort of adult participants with AHD in this project evaluation that started ART from November 2018 to June 2019 and we abstracted data from their medical records; the follow-up period was for 6 months.

The study was conducted at the ART clinics at the Motebang and Berea government hospitals that are managed by the Leribe and Berea District Health Management Teams (DHMT), respectively. These are the two largest district hospitals in their respective districts serving one-third of Lesotho’s population. Motebang hospital provided services to 5,807 HIV-positive patients and Berea served 4,620 HIV-positive patients. From November 2018 to June 2019, we screened all HIV-positive patients and enrolled adults aged ≥15 years who met the WHO criteria of AHD, (i.e. a recent CD4 cell count result of <200 cells/mm^3 ^within six months prior to or ordered at project enrollment or WHO clinical stage 3 or 4) and either ART naïve (e.g., previously diagnosed as HIV-positive but not on ART or newly diagnosed as HIV-positive) or with interrupted ART (not on ART and returned to care 7 or more days after a missed appointment).

### Program enhancements to facilitate the implementation and integration of the AHD package of care

Job aids were developed and introduced to facilitate the delivery of the AHD package of care including an AHD care checklist to be used in the medical charts of all patients identified with AHD. Participants with WHO stage 3 and 4 disease were classified as “critical clinical staging” and participants with CD4 cell counts <200 cells/mm^3^ were classified as “critical immunologic status”. These classifications required immediate action to offer the essential interventions per job aids. In addition, CD4 cell count results of <200 cells/mm^3^ (from AQUIOS machines) were classified as an “urgent lab result” that required laboratory personnel to take immediate action to notify the clinical team of the results. Once a patient was designated as having AHD, a bright removable sticker was placed on the top of the medical chart and inside the personal medical booklet (bukana), which triggered clinic staff to use the AHD job aids and to place the appropriate job aid checklist in the patient’s chart. Participants with AHD were then given priority for consultation when they presented to the ART clinic.

Extensive training of clinical and laboratory personnel on AHD and the AHD care package was undertaken using the new procedures and job aids to ensure all staff understood the need for prompt identification and treatment of patients with AHD. The training included prompt identification and triage of patients presenting with “danger signs” (defined as respiratory rate >30 breaths/minute; temperature > 39°C; heart rate >120 beats/minute; altered mental status [e.g., confusion, strange behavior, reduced level of consciousness by abnormal Glasgow Coma Scale]; any other neurological problem: seizures, paralysis, difficulty talking, cranial nerve problems; rapid deterioration in vision; airway issue [i.e. new or worsening adenitis, with obstructive symptoms]; unable to walk unaided), the importance of referring AHD patients to a higher level of care and ensuring that ART initiation was not delayed while awaiting CD4 cell count results.

#### Procedures

The advanced HIV disease package of care for Lesotho highlighted the key interventions that were critical for PLHIV with advanced disease and promoted the timely receipt of the key interventions by including a suggested time parameter in accordance with the standard of care as endorsed by the MOH. The package included the following interventions and time parameters:

1. TB screening at all visits followed by prompt initiation of TB treatment for those co- infected with TB within three days of positive TB diagnosis and prompt initiation of Isoniazid Preventive Therapy (IPT) for those screening negative for TB signs and symptoms within three days of negative TB screen;
2. Documented clinical examination to evaluate for signs of any OI at each clinical visit or evidence of IRIS;
3. Same-day serum CrAg screening test for all patients enrolling in care with CD4 count <100 cells/mm3 or clinical stage 3 or 4 and prompt initiation of pre-emptive fluconazole for asymptomatic patients with a positive screen and without evidence of disseminated disease (on exam or by lumbar puncture (LP) when available);
4. Same-day initiation of co-trimoxazole (or dapsone) preventive therapy for the prevention of *Pneumocystis jirovecii* pneumonia (PCP), toxoplasmosis, and serious bacterial infections;
5. Rapid initiation of ART within a seven-day timeframe from enrollment in care with note of the few caveats when delaying ART initiation is the standard of care/recommended by WHO (i.e. Patient with HIV/central nervous system (CNS)-TB co-infection, or HIV/CrAg-positive);
6. Intensive follow-up with weekly phone calls for the first four weeks of follow-up and active tracking of missed appointments (made with patient’s verbal consent) with more frequent clinical visits (including a visit at week eight and eighteen) to rescreen for OIs and signs of IRIS);

Village health workers and other cadres tasked with tracking ART clients in the community were trained on new intensified follow-up procedures for PLHIV with advanced disease. Clinic staff were also trained to make weekly calls to clients based on a standard script and telephone findings were documented.

### Data collection and statistical analysis

Facility personnel provided routine care, identified patients with AHD and completed a standardized AHD checklist. Study nurses abstracted relevant clinical and laboratory data from medical records (e.g., HIV testing and counselling services register, ART register, TB detection register, IPT register, TB register, TB treatment card, HIV ART card, drug dispensing log, laboratory result logs, patient chart, and project forms) and data were entered into a Microsoft Access (version 2013) database programmed with logical data validation checks. For patients with danger signs requiring inpatient care, data abstraction was carried out at the time of discharge from the health facility.

Quantitative data analysis was performed using STATA version 15.1 (College Station, TX, USA). Categorical variables were summarized using frequencies and percentages and continuous variables using means (+/- standard deviation). Descriptive analysis was performed to capture patient outcomes. Kaplan Meier was used to estimate the risk of survival over time. Complete case analysis was performed with no missing data imputed.

#### Ethical review

The study was approved and overseen for human subject protection locally in Lesotho by the Lesotho Ministry of Health research and ethics committee (REC) and in the USA by ADVARRA (formerly known as Chesapeake) institutional review board. We requested and obtained waiver of consent for several reasons, all of which conform with United States (US) Federal regulations OHRP-45CFR46.116, DHHS- 45CFR46.117(c), the local IRB requirements and HIPAA-CFR 164.512(i)(2)(ii).

This program evaluation and all the procedures involved with collecting and analyzing routine program data imposed minimal risk to participants because it did not involve direct interaction between the program evaluation staff and HIV care and treatment clients. Routine procedures associated with the implementation of HIV care and treatment guidelines and activities undertaken to strengthen the HIV program did not normally require written consent outside of a research setting. In addition, there was an adequate plan to protect patient identifiers from improper use and disclosure; there was a plan to destroy patient identifiers at the earliest opportunity; and there was written assurances that all health information were protected and were not reused or disclosed to any other person or entity except as required by law. A waiver of consent did adversely affect the rights and welfare of participants because no identifiable information was recorded on CRFs to be entered into the evaluation database or in the evaluation database itself.

## Results

**Fig 1:**
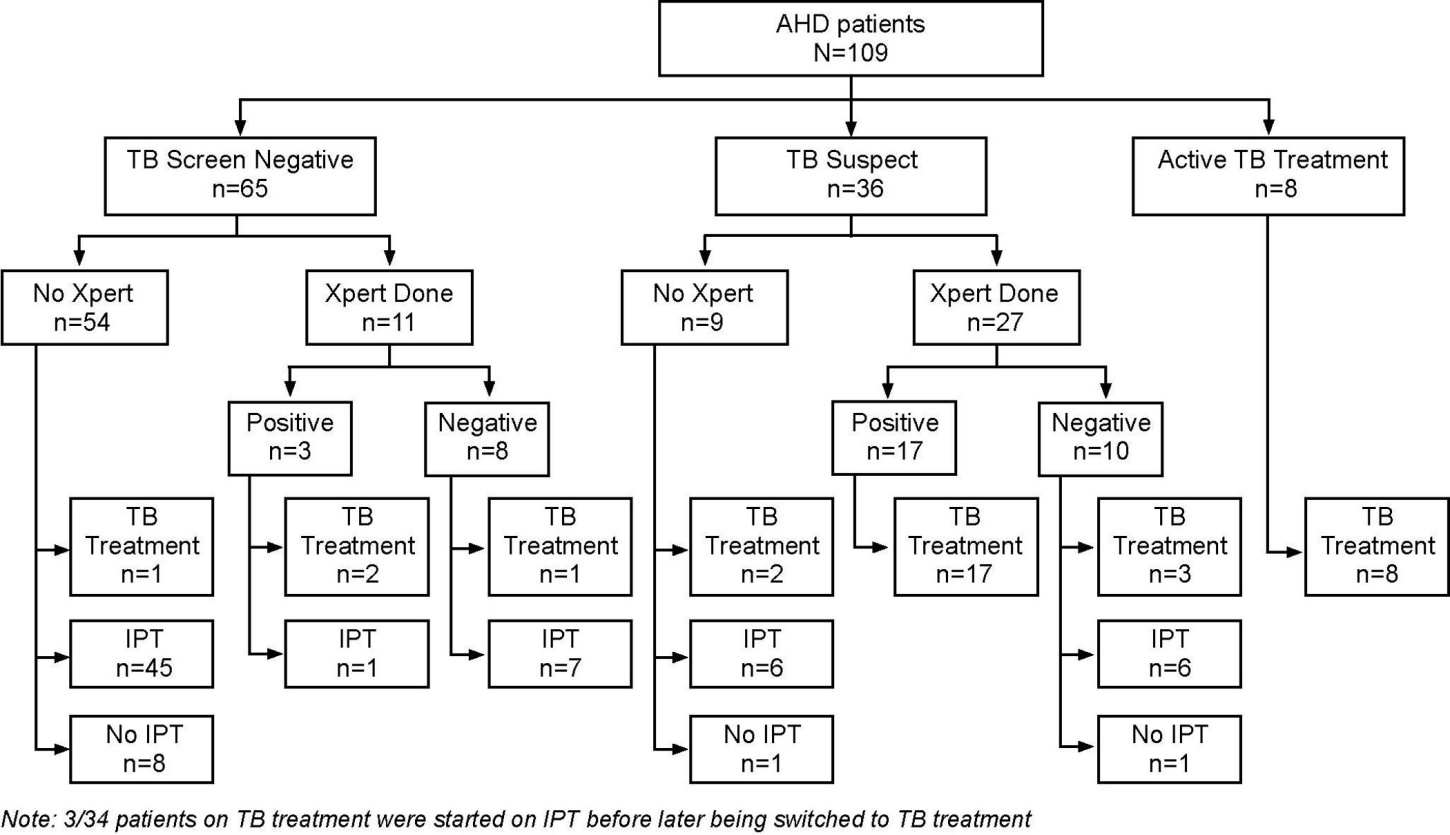
TB screening, diagnosis, prevention and treatment cascade. This figure captures the TB cascade among study participants. This includes symptom-based screening, diagnosis including laboratory, initiation on TB preventive therapy and the treatment initiation and outcomes.

Of the 150 participants identified with AHD, 41 were excluded with the most common reason being patients who opted to register for ART services at a non-study site (Fig 1). We prospectively enrolled 109 patients in this project evaluation (73.2%) where they received the AHD care package. Table 1 displays the baseline demographic and clinical characteristics of enrolled study participants. Among enrolled participants, 62/109 (56.9%) were men, and the overall mean age was 38 (SD = 9.9) years with no significant sex differences by age. Among the 109 participants enrolled, 88 (80.7%) had clinical stage 3 or 4 disease. A total of 72 (66.1%) out of the 109 enrolled participants had CD4 count results, of which 60/72 (83.3%) had AHD by CD4 definition; 37/72 (51.4%) had CD4 <100 cells/mm^3^ and 23/72 (31.9%) had CD4 count result range of 101–199 cells/mm^3^. At the initial clinic visit, 65/109 (59.6%) screened negative for TB based on symptoms, 36/109 (33%) patients had presumptive TB based on positive TB symptom screening, and 8/109 (7.3%) were already on TB treatment (Table 1 and Fig 1). Thirty-eight participants had Xpert MTB/RIF testing (27 TB symptom screen positive and 11 screen-negative cases) with 52.6% (20/38) being confirmed to have TB. Of note was that three participants who were Xpert-positive were originally TB symptom screened negative and 17 Xpert positive were originally TB symptom screened positive. Overall, 34/109 (31.2%) participants were diagnosed with TB. All Xpert positive TB participants except one (19/20, 95.0%) were initiated on TB treatment. Seven participants without a positive Xpert MTB/RIF result were started on TB treatment. The rest of the participants not on TB treatment i.e. 86.7% (65/75) initiated IPT.

**Table 1:** Baseline demographic and clinical characteristics of participants (N = 109).

Characteristic	n (%)
**Sex** Female Male	47 (43.1) 62 (56.9)
**Mean age ± SD (years)**	38.0 ± 9.9
**Age (years)** 15–19 20–24 ≥ 25	3 (2.8) 3 (2.8) 103 (94.5)
**WHO Clinical Stage** 1 2 3 4	11 (10.1) 10 (9.2) 86 (78.9) 2 (1.8)
**CD4 count result (cells/ mm^3^)** <100 101–199 ≥200 Missing	37 (51.4) 23 (31.9) 12 (16.7) 37
**TB screening at baseline** TB symptom screen Negative TB symptom screen positive On TB treatment at enrollment	65 (59.6) 36 (33.0) 8 (7.3)
**Isoniazid preventive therapy initiation during study** Initiated Not initiated Ineligible due to active TB treatment Initiated on IPT, later switched to active TB treatment	65 (59.6) 10 (9.2) 31 (28.4) 3 (2.8)
**CD4 <100 and/or WHO Stage 3 or 4**	97 (89.0)
**CrAg screening status among eligible (n = 97)** Not screened Screened at initial visit Screened during follow-up	
	52 (53.6) 39 (40.2) 6 (6.2)
**Cotrimoxazole initiation at first clinic visit** No^a^ Yes Yes, before first clinic visit Missing	1 (0.9) 81 (74.3.) 26 (23.8) 1 (0.9)
**No prior ART exposure**	102 (93.6)
**ART initiated during study** No Yes, at first clinic visit Yes, after enrollment, before first clinic visit Yes, during study follow-up	6 (5.5) 57 (52.3) 20 (18.3) 26 (23.9)
**Reason for not initiating ART at first visit** Active TB treatment Cryptococcal meningitis treatment Other illnesses Transfer out	n = 32 24 (75.0) 4 (12.5) 3 (9.4) 1 (3.1)

^a^ This patient was initiated on Cotrimoxazole during a study follow-up visit.

**Fig 2:**
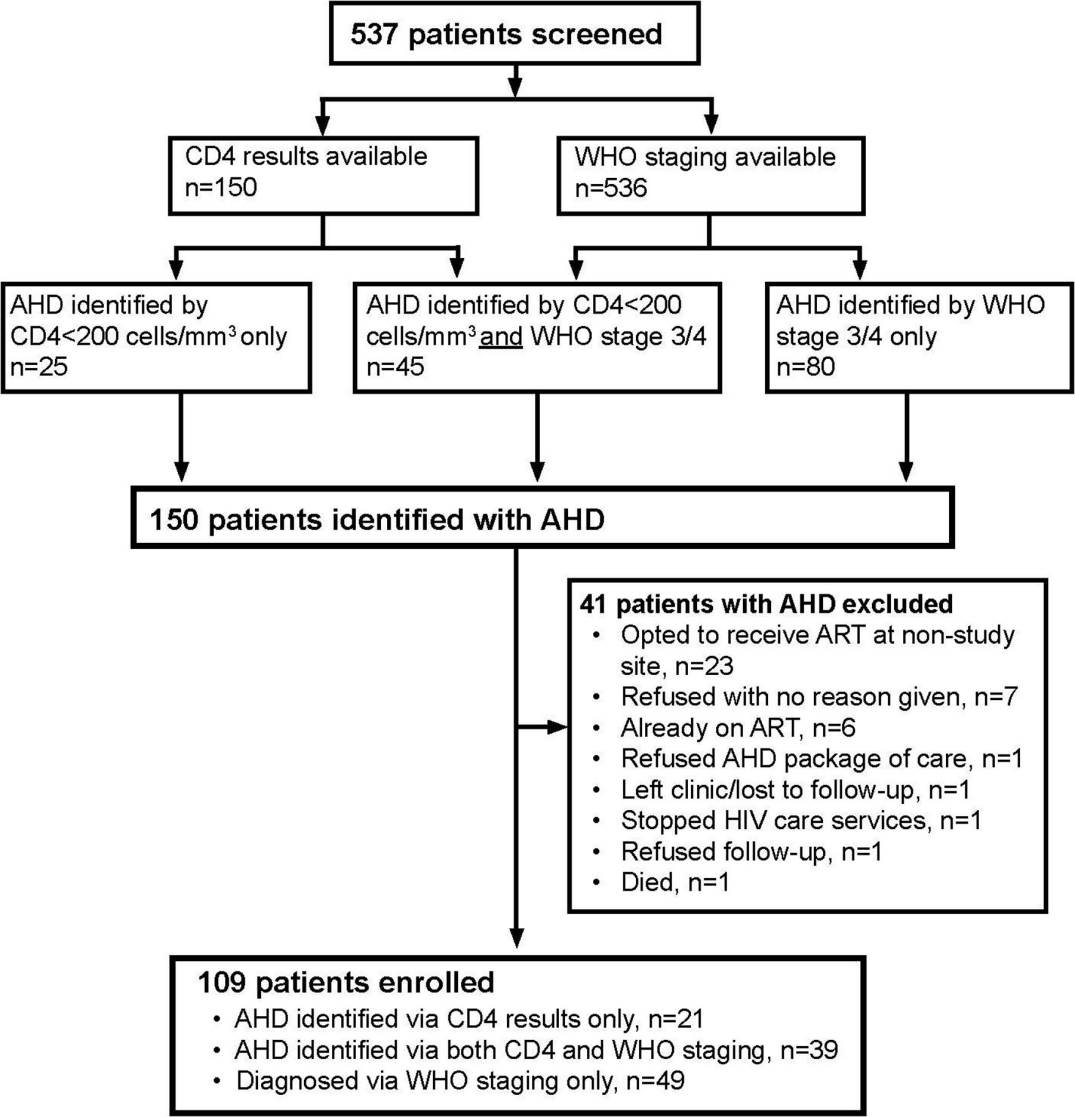
Screening and enrollment of participants. This figure describes the identification of patients with AHD and their enrollment into the study.

Overall, there were 537 HIV-positive participants screened for AHD. Of these participants, 150 (27.9%) had a recent CD4 cell count test result within six months prior to enrollment or ordered at screening and 70/150 (46.7%) had a CD4 count result of <200 cells/mm^3^ (Table 2 and Fig 2). All participants, except one, were clinically staged, with 125/537 (23.3%) WHO stage 3 or 4 and of these, 67 did not have a CD4 count result. Fifty-eight participants had a CD4 count result, and 45/58 (77.6%) had CD4 <200 and 13/58 (22.4%) had CD4 ≥200. Among 91 patients with WHO stages 1 or 2 and CD4 count results at screening, 25 (27.5%) had CD4 <200. Overall, 150/537 (27.9%) participants met the criteria for AHD (80 based on only clinical stage 3 or 4; 25 based on only CD4, and 45 based on both CD4 and clinical staging). Almost all participants with AHD (95%) were 25 years or older. Twenty-one (3.9%) participants presented with danger signs at the time of study screening, of whom 18/21 (85.7%) had AHD.

**Table 2:** Characteristics of HIV-positive participants screened for AHD (N = 537).

Characteristics at Screening	n (%)
**Total patients screened with recent CD4 cell count**	150 (27.9)
**Timing of CD4 cell count result (n = 150)** Within last 6 months prior to screening Same day of screening Within 1 week of screening More than a week post screening Missing	9 (6.0) 118 (79.2) 12 (8.1) 10 (6.7) 1
**CD4 cell count result <200 cells/ mm^3^ (n = 150)**	70 (46.7)
**WHO clinical stage** 1 2 3 4 Missing	314 (58.6) 97 (18.1) 122 (22.8) 3 (0.6) 1
**AHD (CD4 <200 cells/ mm^3^ or stage 3 or 4)**	150 (27.9)
**AHD by age group (n = 150)** 15–19 20–24 ≥ 25 Missing	3 (2.0) 4 (2.7) 141 (95.3) 1
**Danger signs present ^a^**	21 (3.9)

^a^ Danger signs: Respiratory rate >30 breaths/minute; temperature > 39°C; heart rate >120 beats/minute; altered mental status (e.g., confusion, strange behavior, reduced level of consciousness by abnormal Glasgow Coma Scale); any other neurological problem: Seizures, paralysis, difficulty talking, cranial nerve problems; rapid deterioration in vision; airway issue (i.e. new or worsening adenitis, with obstructive symptoms); unable to walk unaided.

A total of 97 participants had CD4 count <100 cells/mm^3^ and/or WHO stage 3 or 4 and were eligible for serum CrAg screening. Among eligible participants, 45 (46.4%) were screened for serum CrAg; 39/97 (40.2%) were screened for serum CrAg at initial visit, and 6/97 (6.2%) were screened during follow-up visits. Among the 45 participants who received CrAg screening 8 (17.8%) were screened based on WHO stage 3 or 4 classification alone and 37 (82.2%) were screened based on CD4 count <100 cells/mm^3^. Among the 39 patients screened at the initial visit, five (12.8%) participants had positive serum CrAg test and had cerebrospinal fluid (CSF) sent for CrAg testing; 4 were confirmed CSF CrAg-positive and diagnosed with CM and initiated on induction treatment with Amphotericin B and fluconazole. The 6 participants screened during follow-up visits were all serum CrAg negative. All but one participant was initiated on cotrimoxazole prophylaxis at their first clinical visit. Overall, 52/97 (53.6%) were not screened for CrAg due to CD4 reagents and CrAg test kit commodity issues.

Among the 109 participants with AHD, 102/109 (93.6%) were ART-naive and 7 reported prior ART exposure, but had stopped taking ART. More than 70% of participants (77/109) initiated ART within 7 days, either before or at their first study visit. Excluding CM and TB cases, 77/81 (95.1%) initiated ART rapidly. A total of 93% initiated (83/89) on a Tenofovir-Lamivudine-Efavirenz (TLE) regimen. Of 32 participants not initiated on ART at their first visit, 24 participants were on active TB treatment, 4 were diagnosed with CM (one CM patient was also later diagnosed with TB) and 3 reported other illnesses (severe diarrhea, feeling weak, esophageal candidiasis, or awaiting Xpert MTB/RIF test result); 26/32 (81.2%) were initiated on ART during the study follow-up with a median ART delay of 14 days.

**Fig 3:**
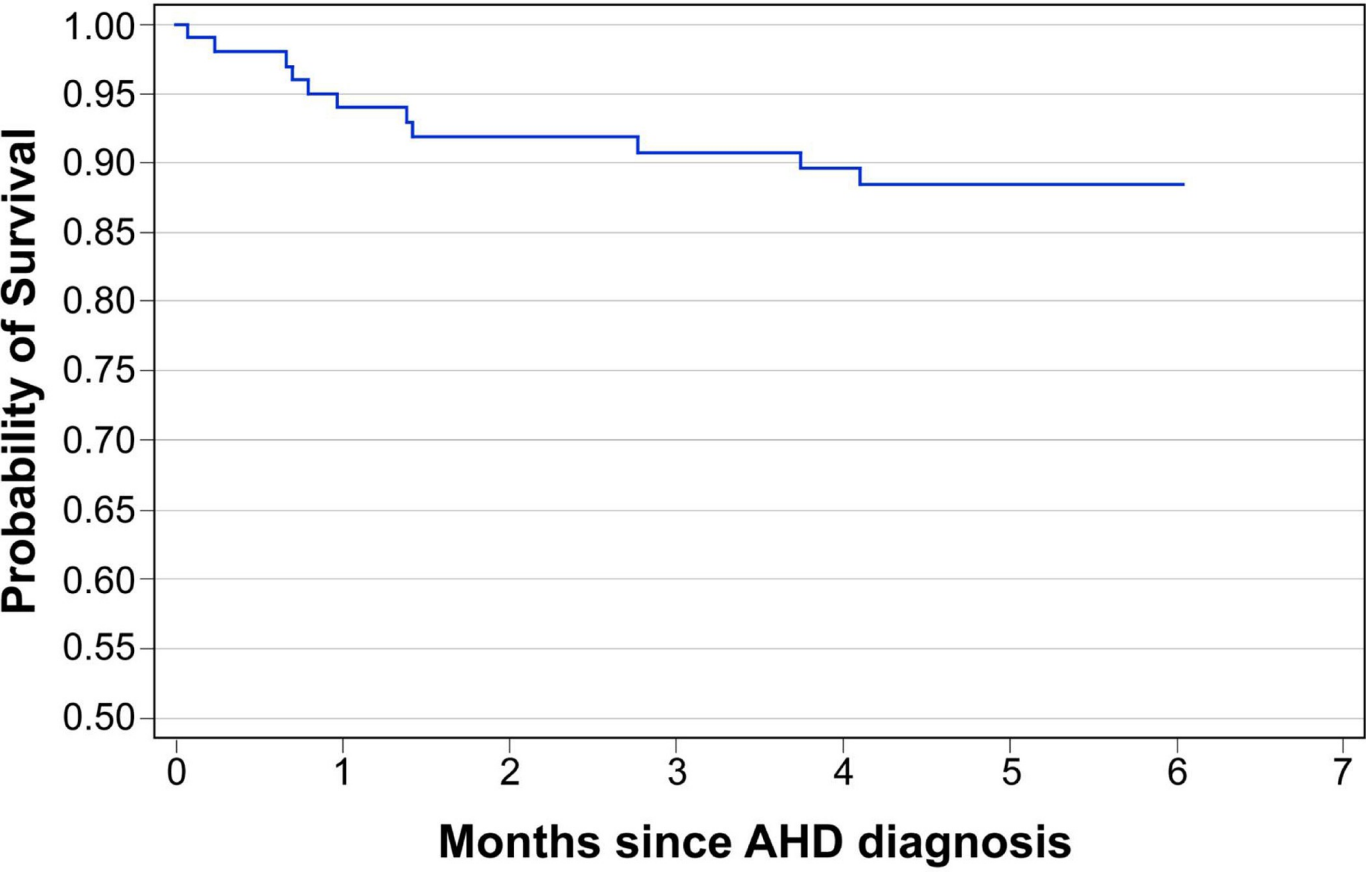
Estimated 6-month survival among patients with Advanced HIV Disease (n = 109). Fig 3 captures the survival analysis over a six-month period from the time of enrollment in the study.

Table 3 summarizes the six-month outcomes among participants with AHD. A total of 76/109 (69.7%) participants completed the study follow-up, 5/109 (4.6%) transferred to non-study health facilities, 17/109 (15.6%) were lost to follow-up, and 11/109 (10.1%) died. Of the 74 participants that completed the study who had an end-of-study viral load (VL) test, 63 (85.1%) were virally suppressed (VL<1,000 copies/ml). Among the 11 participants who died, 3 (27.3%) died before ART initiation, 6 (54.5%) died within three months of ART initiation, and the remaining 2 (18.2%) participants died 3 to 6 months after ART initiation. Nine of the eleven participants who died had TB; one TB patient also had CM at the time of death. The Kaplan Meier estimate of cumulative probability of survival from AHD diagnosis to 6 months was 87.4% (Fig 3).

**Table 3:** Advanced HIV disease outcomes after six months follow up (N = 109).

Outcomes	n (%)
**Final study outcomes at 6 months** Completed study Lost to follow-up Died Transferred out	76 (69.7) 17 (15.6) 11 (10.1) 5 (4.6)
**Viral status at end of study** < 1000 copies/mL ≥ 1000 copies/mL Missing	63 (85.1) 11 (14.9) 2

## Discussion

This first study on AHD implementation in Lesotho in the Test and Treat era found that more than a quarter of newly diagnosed HIV patients (27.9%) were presenting with AHD. The implementation of the AHD package did not delay the rapid initiation of ART among most eligible patients and 98.1% of eligible patient were initiated on ART. Overall implementation of several elements of the package of care was successful, close to 100% received cotrimoxazole and nearly 90% of eligible patients received IPT. CD4 screening proved to be the most challenging aspect of the AHD package implementation with a coverage of only 28%. As less than a third of screened patients with AHD received CD4 testing, over half of eligible participants did not receive CrAg screening. Of importance, all those who were diagnosed with cryptococcal meningitis were initiated on the recommended treatment and survived during the six months of study follow up except one that had multiple pathologies including TB. In addition, retention and viral suppression rates were at 85%. Our results highlight the feasibility of implementing a differentiated care model for PLHIV with AHD with favorable outcomes of retention and viral load suppression (VLS) for this population.

Most participants enrolled in this study were initiated on ART in a timely fashion with reasons for ART initiation delay due to diseases such as TB, cryptococcal infection and a small number of patients who were still under investigation for other diseases. Therefore, there were very few missed opportunities for rapid ART initiation. As per the national guidelines at the time, almost all patients in our study were initiated on TLE at a rate similar to the general ART population, which ranges from 85–95% from program data and other concurrent studies from Lesotho [18]. We observed mortality of 10.1% in AHD patients enrolled to receive the WHO package for AHD. Although there is no documentation of baseline mortality among AHD patients in Lesotho, other studies from the region have reported comparable results. The REALITY trial that included patients starting ART with a CD4 count <100 cell/mm^3^, reported a mortality of 12.2% at 24 weeks and 14.4% at 48 weeks among patients who received standard prophylaxis [9]. More recently, 9.5% mortality at 12 months of follow-up was reported in a Rwandan cohort of patients with AHD [19]. In our study, most of the participants who died were diagnosed with active tuberculosis. This is not surprising because TB is the most common co-infection among positive patients. The leading cause of death in the REALITY study was unknown followed by TB [20]. Nine out of the eleven patients who died in our study were diagnosed with TB. This underscores the need for rapid identification of TB co-infection and treatment of these patients. Our results provide additional evidence supporting early initiation of ART for co-infected patients and agree with the 2021 revision of WHO guidelines. The guidelines have been adopted by the Lesotho government. Aggressive TB preventive therapy programs using drugs such as Isoniazid in AHD patients have been well described [21]. Similar to our study where we found TB-coinfection prevalence of 31.2%, many settings have shown high prevalence and severity of TB co-infection among HIV-positive persons with AHD [22–24]. Our finding of a high TB prevalence is consistent with the 2019 Lesotho TB Prevalence study, which noted a high TB prevalence (581 per 100,000 (95% CI: 466–696) in the adult population ≥15 years of age and even higher among those with an HIV positive status (1,272 per 100,000) [25]. Over 85% of patients eligible for IPT received it. While the study was not designed to assess IPT initiation as an intervention, IPT remains an important component of the package of care for PLHIV.

The VLS among AHD patients in our study was 85.1%, at six month-follow up. The 2016–2017 Lesotho Population-based HIV Impact Assessment (LePHIA) conducted shortly before our study and the 2020 LePHIA reported VLS of adults on ART of 87.7% and 92%, respectively [26,27].

Considering the mixed characteristics of a general population [28] and vulnerability of AHD patients [3], the slightly lower VLS rate than the general population was not unanticipated and leaves room for programmatic improvement. Accounting for deaths, LTFU, and transfer-outs, our study completion of 69.7% represented a much higher attrition than the 82.3% reported from a similar cohort in Zimbabwe [29]. This underscores the need for design and implementation of robust strategies for patient follow-up at facility and community level [30]. Our findings demonstrate that with provision of the recommended WHO package for AHD, VLS outcomes and survival may reach levels similar to that of the population without AHD, but retention efforts may need additional attention and interventions. However, since the completion of our study, Lesotho has aggressively implemented Dolutegravir-based regimens for all PLHIV [31], which will likely further improve VL suppression and other key health outcomes in PLHIV, including those with AHD [32].

Despite implementation of the Test and Treat approach, more than a quarter of patients who presented in the study sites had AHD with more than fifty-six percent of whom were male. These are important findings because it was expected that scale-up of Test and Treat would significantly reduce the proportion of patients who present with AHD. Similar to these findings, several studies in South Africa found AHD prevalence of 32.9% in 2016, 21.8% in 2017 while in Ethiopia, Lifson et al found that 60% of patients presented with AHD between 2015 and 2017 [33–35]. Most recent data show the persistence of AHD as demonstrated in a study of Southern African countries (Lesotho, Malawi, Mozambique, South Africa, Zambia, and Zimbabwe), which noted that the percentage starting ART with AHD (CD4 <200) declined from 83.3% in 2005 to 23.5% in 2018 and this was concurrent with evolving WHO ART initiation guidelines and in the later years lower rates of CD4 testing and shorter time from HIV diagnosis to ART initiation [36,37]. Similarly, high rates of men presenting with AHD has been observed in many of these countries [10,34,35,38]. How optimized ART will impact AHD is yet to be seen, but issues of stigma and early HIV case identification challenges may continue to contribute to PLHIV presenting to care and treatment late. These findings and considerations demonstrate the ongoing need to rapidly identify and diagnose PLHIV with AHD and scale-up implementation of the AHD package.

Due to a shortage of CD4 reagents at the beginning of the study, CD4 testing could not be performed systematically for all patients screened which led to a reliance on clinical staging for identifying AHD. Over one-third of participants in our study with CD4<200 had clinical stage 1 or 2 disease. Several other studies have shown that clinical staging does not accurately predict immunological deterioration [6,9–11]. In the REALITY trial, nearly half of those with CD4 <100 had clinical stage 1 or 2 disease [9]. In addition, study sites could not perform routine serum CrAg screening due to CD4 reagent and CrAg test kit stock outs. Despite training providers to perform serum CrAg screening for patients with clinical stage 3 or 4 disease, we found that providers did not readily offer this screening when CD4 testing was not available (i.e. due to reagent stock out and/or CD4 machine breakdown). These findings bolster the call and need for reliable CD4 testing. Finally, there were periodic stock outs of Isoniazid, the current drug used for TPT in Lesotho causing delayed initiation, along with challenges in commodities impacting Xpert MTB/RIF testing. Health systems’ strengthening, including improved communication between health facility clinicians, the district pharmacist, and the supply chain management team at the district, was achieved throughout the study as the implementation progressed. These efforts led to more accurate commodity forecasting and subsequent reduction in stock-outs of commodities so that most diagnostics and drugs were consistently available in the study sites. However, the stock-out challenge experienced at the start of the study highlighted the realities field health care workers experienced in putting into practice AHD guidelines and policies.

Our study had some limitations. The study occurred in two sites that were purposively selected. These sites enrolled adults from rural and/or urbans areas. Several patients had AHD but were not enrolled because they opted to register for ART services elsewhere. We did not enroll patients currently on ART with AHD. Although the study findings may not be generalizable to pediatric patients given the youngest age band included was adolescents 15–19 years old, they still contribute to understanding the magnitude of AHD and associated comorbidities in the Test and Treat era. Another limitation is the impact of the stock-out of commodities in the context of program implementation; it is likely that the stock-out of CD4 commodities led to an underestimation of the AHD prevalence and less CrAg screening. Newer, rapid diagnostics tests for AHD diagnosis (e.g., semi-quantitative lateral flow CD4 and lateral flow urine TB lipoarabinomannan assay) were not yet available in Lesotho at the time of our study. Despite the above challenges, the observed study outcomes in this program context suggest the feasibility and scalability of our results in routine HIV clinical settings since the study took place in the real program setting.

Ultimately, the Lesotho Ministry of Health, along with stakeholders have since taken the results of this AHD evaluation to inform the development of the National AHD Guidelines [39]. AHD services are now offered in hospitals across the country and AHD progress is reported and monitored.

## Conclusions

Our study found a high rate of AHD in Lesotho three years after the implementation of the Test and Treat policy. The implementation of a differentiated model of care for patients with AHD did not prevent rapid ART initiation among those with no contraindications and most of the components of the AHD package were feasible to implement. We found VLS of more than 80%, which was comparable and within reach of the general population on ART. Optimal retention strategies and use of newer, rapid diagnostic tests for AHD need further exploration. While the overall survival rate was high, the leading cause of death was TB.

We recommend rapid adoption of the AHD guidelines and scale-up of the AHD package and implementation of a differentiated service model for patients with AHD in other districts. This must include optimized point of care diagnosis for TB and rapid initiation of TB-HIV co-infected patient on treatment. Commodities security and health system optimization efforts are vital components for AHD and necessary for the successful diagnosis and management patients at high risk for morbidity and mortality.

## Supporting information

S1 File (DOCX)
